# SIRT3 ameliorates diabetes-associated cognitive dysfunction via regulating mitochondria-associated ER membranes

**DOI:** 10.1186/s12967-023-04246-9

**Published:** 2023-07-22

**Authors:** Yanmin Chang, Cailin Wang, Jiahui Zhu, Siyi Zheng, Shangqi Sun, Yanqing Wu, Xingjun Jiang, Lulu Li, Rong Ma, Gang Li

**Affiliations:** 1 Department of Neurology, Union Hospital, Tongji Medical College, Huazhong University of Science and Technology, Wuhan, 430022 China; 2 Department of Pharmacology, School of Basic Medicine, Tongji Medical College, Huazhong University of Science and Technology, Wuhan, 430030 China

**Keywords:** Diabetes-associated cognitive dysfunction, Sirtuin3, Mitochondria-associated ER membranes, VDAC1–GRP75–IP3R complex, Honokiol

## Abstract

**Background:**

Diabetes is associated with an increased risk of cognitive decline and dementia. These diseases are linked with mitochondrial dysfunction, most likely as a consequence of excessive formation of mitochondria-associated membranes (MAMs). Sirtuin3 (SIRT3), a key mitochondrial NAD^+^-dependent deacetylase, is critical responsible for mitochondrial functional homeostasis and is highly associated with neuropathology. However, the role of SIRT3 in regulating MAM coupling remains unknown.

**Methods:**

Streptozotocin-injected diabetic mice and high glucose-treated SH-SY5Y cells were established as the animal and cellular models, respectively. SIRT3 expression was up-regulated in vivo using an adeno-associated virus in mouse hippocampus and in vitro using a recombinant lentivirus vector. Cognitive function was evaluated using behavioural tests. Hippocampus injury was assessed using Golgi and Nissl staining. Apoptosis was analysed using western blotting and TUNEL assay. Mitochondrial function was detected using flow cytometry and confocal fluorescence microscopy. The mechanisms were investigated using co-immunoprecipitation of VDAC1–GRP75–IP3R complex, fluorescence imaging of ER and mitochondrial co-localisation and transmission electron microscopy of structural analysis of MAMs.

**Results:**

Our results demonstrated that SIRT3 expression was significantly reduced in high glucose-treated SH-SY5Y cells and hippocampal tissues from diabetic mice. Further, up-regulating SIRT3 alleviated hippocampus injuries and cognitive impairment in diabetic mice and mitigated mitochondrial Ca^2+^ overload-induced mitochondrial dysfunction and apoptosis. Mechanistically, MAM formation was enhanced under high glucose conditions, which was reversed by genetic up-regulation of SIRT3 via reduced interaction of the VDAC1–GRP75–IP3R complex in vitro and in vivo. Furthermore, we investigated the therapeutic effects of pharmacological activation of SIRT3 in diabetic mice via honokiol treatment, which exhibited similar effects to our genetic interventions.

**Conclusions:**

In summary, our findings suggest that SIRT3 ameliorates cognitive impairment in diabetic mice by limiting aberrant MAM formation. Furthermore, targeting the activation of SIRT3 by honokiol provides a promising therapeutic candidate for diabetes-associated cognitive dysfunction. Overall, our study suggests a novel role of SIRT3 in regulating MAM coupling and indicates that SIRT3-targeted therapies are promising for diabetic dementia patients.

**Supplementary Information:**

The online version contains supplementary material available at 10.1186/s12967-023-04246-9.

## Introduction

Diabetes-associated cognitive dysfunction has become a common and serious health concern worldwide [1–3]. Previous studies have indicated that hyperglycemia or high glucose (HG), a pathologic factor in type 1 diabetes, induces significant neuronal dysfunction and cognitive impairment in both human and animal models [4–6]. Mitochondrial dysfunction has been linked to increased oxidative stress and impaired energy metabolism, which plays a critical role in Alzheimer’ disease-type pathologies preceding the appearance of plaques and tangles [7]. Abnormalities in mitochondria-associated endoplasmic reticulum (ER) membranes (MAMs), the physical structures mediating the communication between the ER and mitochondria, has been identified as a chief culprit of mitochondrial dysfunction.

Physiologically, MAM fluctuate dynamically to regulate cellular function, such as autophagy, mitochondrial dynamics, and lipid and calcium trafficking between the mitochondria and ER [1, 8, 9]. These two organelles are tethered by several molecular components of the MAM fraction. One of the most well-characterized macromolecular complexes of MAM is the tripartite complexes, of which cytoplasmic chaperone glucose-regulated protein 75 (GRP75) tethers ER-resident inositol 1,4,5-trisphosphate receptor (IP3R) to the mitochondria-localised voltage-dependent anion channel 1 (VDAC1) [10]. Notably, excessive MAM formation is known to trigger the cascade of pathological events, such as mitochondria calcium overload, aberrant lipid levels, autophagosome formation, and eventually, cell apoptosis [11–14]. However, the mechanisms underlying the induction of MAMs dysfunction by hyperglycemia in diabetes-associated cognitive dysfunction remain unknown.

Sirtuin3 (SIRT3) is a nicotinamide adenine dinucleotide NAD^+^-dependent deacetylase. It is primarily located in the mitochondria and maintains metabolic adaptations [15, 16]. Previous studies have proposed that the loss of function of SIRT3 causes significant mitochondrial dysfunction and oxidative stress [17–19]. Contrarily, the gain of function of SIRT3 can limit high levels of mitochondrial damage and oxidative stress, which in turn prevents apoptosis [20, 21]. Recent studies have demonstrated that the down-regulation of SIRT3 leads to the development of several diseases, such as neurodegenerative diseases, cancers, and heart disease [22, 23]. However, whether SIRT3 exerts a critical role in MAM coupling and diabetes-associated cognitive dysfunction remains elusive.

In this study, we investigated the role of SIRT3 in hippocampal injuries and cognitive impairment in diabetic mice and cell injuries in HG-induced SH-SY5Y cells. We also explored its potential association with aberrant MAM formation. Collectively, our findings revealed the protective role of SIRT3 against diabetes-associated cognitive decline, making it a novel therapeutic candidate for the treatment of diabetes-related cognitive diseases.

## Material and methods

### Cell culture and lentiviral infection

Human neuroblastoma SH-SY5Y cells were cultured in Dulbecco’s modified Eagle’s medium (DMEM; Gibco, United States) containing normal glucose (NG, 5.5 mM), supplemented with 10% fetal bovine serum at 37 °C in humidified 5% CO_2_ and 95% air. When reached 60–80% confluency, cells were switched to serum-free DMEM containing 5.5 mM d-glucose. High glucose (HG, 25 mM) treatment was performed by treating cells with serum-free DMEM containing 25 mM D-glucose for 48 h. A recombinant lentivirus vector (pSLenti-EF1-EGFP-P2A-Puro-CMV-MCS-SIRT3-3xFLAG-WPRE), purchased from OBiO (Shanghai, China), was used for SIRT3 overexpression.

### Isolation of mitochondrial and cytosolic fractions

Mitochondrial and cytosolic fractions were isolated from SH-SY5Y cells via differential centrifugation using the Mitochondria Isolation and Protein Extraction Kit (Proteintech, Wuhan, China). All fractionation steps were conducted at 4 °C. In brief, the cells sample were homogenised in solution A, and the homogenate was added gently to the top layer of an equal volume of solution B. The mixture was centrifuged at 600*g* for 10 min to remove nuclei and unbroken cells. The supernatant was collected and further centrifuged at 10,000*g* for 10 min. After centrifugation, the supernatant was transferred to another centrifuge tube and designated as the cytoplasm fraction. Whereas, the pellet was resuspended in a lysis buffer and used as the crude mitochondrial fraction.

### Western blotting

Cells were collected and incubated with RIPA lysis buffer on ice for 10 min, and the sections of mouse hippocampal cornu ammonis 1 (CA1) were mechanically homogenised in RIPA buffer. After centrifugation for 15 min at 12,000*g* and 4 °C, supernatants containing proteins were mixed with SDS loading buffer and boiled for 10 min. The protein concentration was measured using a BCA kit (Beyotime, Shanghai, China). Denatured protein samples were separated via electrophoresis on a SDS-PAGE gel and transferred to nitrocellulose membranes. Blots were blocked with 5% non-fat milk for 1 h at room temperature and then incubated with primary antibodies overnight at 4 °C. After washing several times in Tris-buffered saline and Tween20 (TBST) under shaker conditions, the membranes were incubated with an appropriate secondary antibody for 1 h at room temperature. Enhanced chemiluminescence (ECL) was then detected using a chemiluminescence detection kit (Biosharp, Anhui, China). ECL Imaging System (610007-8Q, Clinx Science Instruments Co., Ltd.) was used for the visualization of protein bands. Quantitative analysis of blots was performed using ImageJ software (Fiji). Information on reagents and antibodies used in this study were listed in Additional file 1.

### Co-immunoprecipitation assay

For co-immunoprecipitation, cells were harvested, incubated in RIPA lysis buffer on ice for 30 min and disrupted via ultrasonication [24]. The mice brain subset hippocampal CA1 was separated and mechanically homogenised in a lysis buffer for immunoprecipitation. Next, the lysates were centrifuged at 14,000*g* for 20 min. Samples containing 2 mg protein were incubated sequentially with 4 μg of anti-VDAC1 polyclonal antibody overnight at 4 °C and then with 50 μL of protein A/G agarose beads (Beyotime) for 4–6 h at 4 °C. Next, beads were collected by and washed thrice with cold PBS. Precipitated proteins were resuspended in 2 × SDS-PAGE buffer and boiled for 10 min. The collected protein sample was analysed via western blotting.An amount of 2 mg of proteins from supernatant lysates was incubated with 4 μg anti-VDAC1 polyclonal antibody overnight at 4 °C, and then incubated with 50 μL protein A/G agarose beads (Beyotime) for 4–6 h at 4 °C. Next, beads were collected by centrifugation and washed three times with cold PBS. Precipitated proteins were resuspended in 2 × SDS-PAGE buffer and boiled for 10 min. The collected protein sample was analysed by western blotting.

### Measurement of ER and mitochondrial co-localisation and mitochondrial membrane potential via confocal fluorescence microscopy

We evaluated the ER and mitochondrial co-localisation using confocal microscopy. For this, SH-SY5Y cells were seeded in 35-mm laser confocal dishes and labelled with 100 nM Mito-Tracker Red CMXRos (Beyotime, Shanghai, China) and 1 μM ER-Tracker Blue-White DPX (Yeason, Shanghai, China) at 37 °C for 30 min. Dual-colour images were captured using a confocal microscope (LSM780, Carl Zeiss Microscopy Cambridge, MA), and mitochondria–ER co-localisation was assessed using Mander’s overlap coefficient, which is commonly used to quantify the degree of co-localisation [25]. Mitochondrial membrane potential (MMP) was measured using tetramethyl rhodamine methyl ester (TMRM; Beyotime, Shanghai, China) according to the manufacturer’s instructions. TMRM signals were captured using an LSM780 laser scanning confocal microscope (Zeiss), and fluorescence intensities were quantified using Zen Blue software (Zeiss). The images were quantified by two independent investigators blinded to the groups.

### Flow cytometric analysis of Mitochondrial Calcium and mtROS

The rhodamine-2 acetoxymethyl ester (Rhod-2/AM) fluorescent probe was used to detect the mitochondrial calcium content as described before [13]. Briefly, SH-SY5Y cells were seeeded in 6-well plates and loaded with 5 μM Rhod-2 AM (Yeason, Shanghai, China) in Hank’s buffered salt solution (HBSS) for 30 min at 37 °C. After rinsing with PBS to remove excess or non-specific probes loaded in mitochondria, cells were incubated further for 30 min to allow complete de-esterification of intracellular AM esters. Then, cells were collected and gently resuspended in 200 μL PBS for flow cytometry. To determine the mtROS production, cells were loaded with 2.5 μM MitoSOX (Thermo Fisher Scientific, United States) for 10 min at 37 °C, washed with PBS, collected, and resuspended in PBS. The loaded cells were then analysed using a Sony ID7000 Spectral Analyser (Sony Biotechnology, Tokyo, Japan). MMP and ROS generation rate are expressed as the proportion (%) of cells with high fluorescence emission intensity.

### Structural analysis of MAMs via transmission electron microscopy (TEM)

Cell and tissue samples were fixed with 2.5% glutaraldehyde at 4 °C overnight and then post-fixed with 1% osmium tetroxide for 2 h. After rinsing with PBS buffer, the samples were dehydrated using an ethanol gradient, infiltrated with 100% propylene oxide, and embedded in epoxy resin for 24 h. Ultrathin sections (50 nm) were prepared and mounted on copper grids. ER-mitochondria contacts were imaged on a TEM (Thermo Fisher Scientific, United States). The distances between ER and mitochondria were analysed using Image J, as previously reported [26, 27]. The distance between both organelles was measured for each 10 nm along the interface between the ER and mitochondria. ER length adjacent to mitochondria was measured where the distance was less than 30 nm.

### Cell viability assay

Cells were seeded in 96-well plates at a density of 5 × 10^3^ cells/well and treated with HG for 48 h. Viable cell counts were measured using the Cell Counting Kit 8 (CCK8). In brief, 10 μL of CCK-8 solution was added into each well and incubated for 1 h at 37 °C. The optical density of each well was measured using a microplate reader at 450 nm.

### Animals and drug administration

All animal experiments were reviewed and approved by the Ethics Committee of Tongji Medical College, Huazhong University of Science and Technology. Wild-type C57BL/6J male mice were obtained from Beijing Vital River Laboratory Animal Technology Co., Ltd. The mice were housed under standard laboratory conditions under an artificial 12-h/12-h light/dark cycle.

To induce chronic hyperglycemia in mice, 2-month-old mice were administered intraperitoneal injections of streptozocin (STZ; 75 mg/kg/day. Solarbio, China) dissolved in citrate buffer (0.1 M, pH = 4.5) for 3 consecutive days, while the control group was administered an equal volume of citrate buffer without STZ [13]. STZ-injected mice were given 10% sucrose water during the following 7 days after STZ administration to prevent low-glucose shock. Seven days later, blood was collected from the tail vein and the glucose level was measured. Mice with a nonfasting blood glucose level of > 16.7 mmol/L were considered diabetic and used in the following experiments. Honokiol (MedChemExpress, United States) was administered at a dose of 40 mg/kg/day for 8 weeks via intraperitoneal injection in diabetic mice [28].

### Stereotactic brain injection

An adeno-associated virus (AAV)-SIRT3 or control virus were bilaterally injected into the hippocampus CA1 region of wild-type mice and STZ-injected mice, respectively. The mice were anesthetized by intraperitoneal injection with a mixture of ketamine (75 mg/kg) and medetomidine (1 mg/kg) and then fixed in a stereotaxic apparatus. A small craniotomy was created at the projecting point of the CA1 region [− 1.94 mm anteroposterior (AP)] relative to the bregma, ± 1.2 mm mediolateral (ML), and − 1.6 mm dorsoventral (DV)]. The virus was injected bilaterally into the hippocampus CA1 region of mice at a speed of 0.1 μL/min with a Hamilton needle by using an automatic microinjection system (World Precision Instruments). The needle was left at the injection point for 10 min before slowly retracting to ensure proper diffusion of the virus. Behavioural assays were conducted 2 months later.

### TUNEL staining

TUNEL staining assay was performed using the One-Step TUNEL in situ Apoptosis Assay Kit (Elabscience, Wuhan, China) according to the manufacturer’s instructions. In brief, paraffin-embedded mouse brain slices (4 μm thick) were deparaffinised in xylene, rehydrated in gradient ethanol, incubated with TUNEL reagent mixture for 60 min, counterstained with DAPI for 5 min and examined under a fluorescence microscope (SV120, OLYMPUS, Japan). The TUNEL index (apoptosis rate) was calculated as the proportion (%) of TUNEL-positive neurons among DAPI-positive neurons.

### Golgi staining and spine analysis

Golgi staining was performed by using an FD Rapid GolgiStain Kit (FD neurotechnology, Beijing, China) as described previously [29]. In brief, the mouse brains were separated and sequentially incubated with the staining buffers A, B, C, D, and E following the manufacturer’s instructions. The brain samples were sliced into 100-μm thick sections using a Vibratome (VT 1000 s, Leica, Nussloch, Germany) and imaged using an optical microscope (Nikon, Japan). Spine density was analysed using ImageJ and expressed as number of spines per 10 μm of dendritic length.

### Nissl staining

Paraffin-embedded brain sections (4-μm thick) were stained with Nissl staining solution (Servicebio, Wuhan, China) for 5 min and then washed with running water until they turned colourless. If the slice was hyperchromatic, 0.1% glacial acetic acid was used for differentiation. The sections were dried, mounted on coverslips using Permount TM Mounting Medium and examined under a scanning microscope (SV120, OLYMPUS, Japan).

### Behavioural tests

After the animals received virus injection or honokiol administration, their cognitive capacity was evaluated by the novel object recognition (NOR) and the Morris water maze (MWM) tests. Tests were conducted as before. The NOR test was conducted to measure learning and memory based on the fact that the mice are born curious. The MWM test was used to evaluate spatial learning and memory.

### Statistical analysis

The distribution normality was tested using the Shapiro–Wilk test. Normally distributed data are presented as the mean ± standard deviation (SD) or mean ± standard error of mean (SEM). Student’s unpaired t-test was used for the comparing the two groups. Differences within the groups were analysed using one-way analysis of variance (ANOVA) followed by Dunnett’s post hoc test. Non-normally distributed data (distance between the mitochondria and ER) are presented as median and interquartile ranges. For evaluating the significance, the Kruskal–Wallis test was used for multiple group comparisons and the Mann–Whitney test was used for two group comparisons. A *p-*value of < 0.05 was considered statistically significant. Statistical analysis was performed using Prism 9.0 GraphPad software.

## Results

### Decreased SIRT3 expression in the hippocampus of diabetic mice and HG-exposed SH-SY5Y cells

**Fig. 1:**
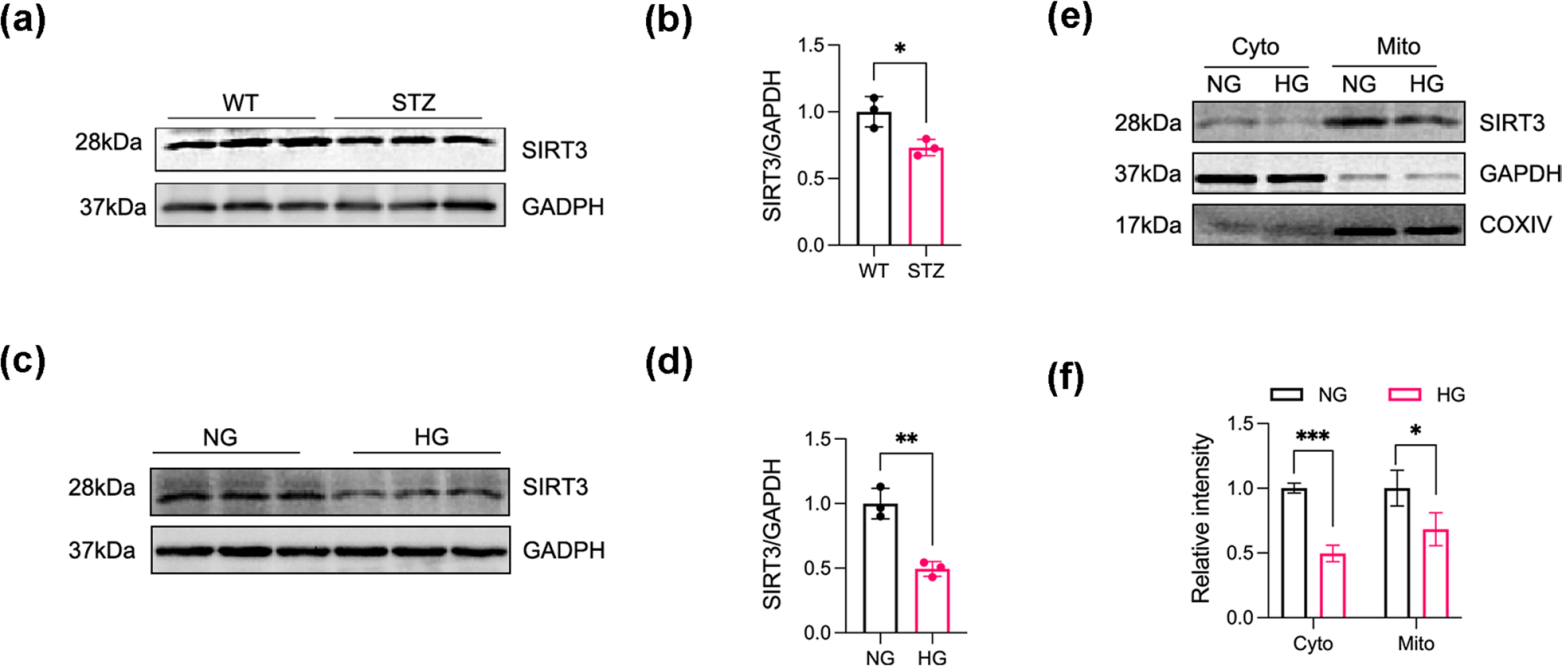
Decreased SIRT3 expression in the hippocampus of diabetic mice and HG-exposed SH-SY5Y cells. **a**, **b** Western blot analysis showing SIRT3 protein expression in hippocampal tissue. N = 3 mice per group. **c**, **d** Western blot analysis of SIRT3 protein expression in SH-SY5Y cells. GAPDH was used as the loading control. N = 3. **e**, **f** Western blot analysis of SIRT3 protein expression in cytoplasmic and mitochondrial fractions extracted from SH-SY5Y cells. Cyto, cytoplasm; Mito, mitochondria. COXIV served as the mitochondrial marker and GAPDH served as the cytoplasmic marker. N = 3. Data were expressed as mean ± SD, *p < 0.05, **p < 0.01, ***p < 0.001

Metabolic disturbances in diseases usuall affect several biochemical mediators. Therefore, we measured SIRT3 expression in the hippocampus CA1 region of the control and STZ-induced diabetic mice. SIRT3 expression levels were significantly lower in the diabetic mice than in the control mice (Fig. 1a, b). Consistent with the animal models, SIRT3 protein levels were drastically reduced in HG-treated SH-SY5Y cells (Fig. 1c, d). To evaluate the subcellular localisation of SIRT3, cytoplasmic and mitochondrial fractions were isolated from SH-SY5Y cells and and subjected to western blotting, which demonstrated that SIRT3 was distributed in in both compartments under resting conditions, but mainly in the mitochondria (Fig. 1e, f). Although HG stimulation decreased SIRT3 expression levels in both the mitochondria and cytoplasm, this decline was more pronounced in the cytoplasm than in the mitochondria, possibly due to the compensatory role of SIRT3 translocated from the cytosol to the mitochondria to maintain cellular function under HG conditions.

### SIRT3 ameliorates hippocampal injuries and cognitive deficits in diabetic mice

**Fig. 2:**
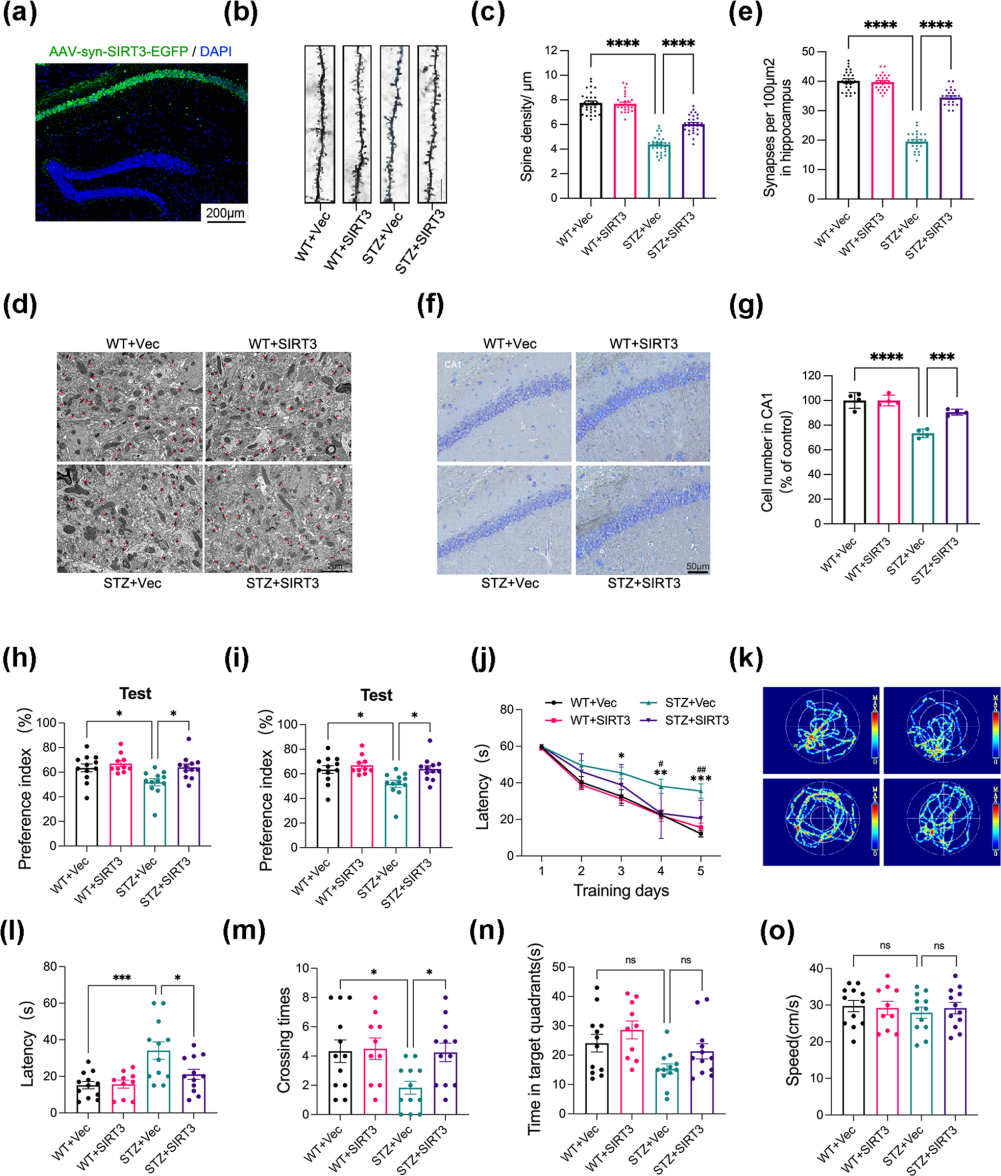
SIRT3 ameliorates hippocampal injuries and cognitive deficits in diabetic mice. **a** Representative fluorescence image confirming the efficiency of virus infection. pAAV-hSyn-SIRT3-3xFLAG-P2A-EGFP-WPRE or the empty vector was stereotaxically infused into the hippocampi of two-month-old mice. After a month, the expression of AAV-SIRT3 was confirmed by fluorescence microscopy. **b**, **c** Representative images and quantitative analysis of spine density in the hippocampal CA1 region of mice measured by Golgi staining. Density is expressed as spine number per 10 μm of dendritic length. Scale bar = 10 μm. N = 30 neurons from 3 mice for each group. **d**, **e** Synaptic density in the hippocampus of mice detected by electron microscopy. N > 25 images from 3 mice for each group. **f**, **g** Representative Nissl staining images and quantitative analysis of Nissl-positive hippocampal neuron number in the CA1 region. N = 3 mice/group. **h**, **i** Preference index during training and testing phases of the novel object recognition (NOR) test. N = 10–12 mice/group. **j**–**o** Spatial learning and memory as measured using the Morris water maze (MWM) test. Latency to find the hidden platform during 5 consecutive training days (**j**). *p < 0.05, **p < 0.01, WT + Vec vs STZ + Vec; #p < 0.05, STZ + Vec vs STZ + SIRT3. Representative swimming paths of each group during the test phase (**k**). Escape Latency to reach the absent platform (**l**). Absent platform crossing frequency (**m**), duration in the target quadrant (**n**) and average swimming speed during the probe test on the 7th day (**o**). N = 10–12 mice/group. Data were presented as mean ± SEM, *p < 0.05, ***p < 0.001, ****p < 0.0001

To reverse SIRT3 down-regulation in diabetic mice, we injected AAV–SIRT3–eGFP into the hippocampal CA1 region on of 2-month-old mice. After a month, the efficiency of virus infection was confirmed by fluorescence image (Fig. 2a). We next examined if SIRT3 overexpression can rescue spine impairments and neuronal loss in diabetic mice. Western blotting of synaptic proteins showed that diabetes reduced the expression of the presynaptic protein Synaptophysin (SYP) and the postsynaptic protein PSD 95, while overexpression of SIRT3 promoted the expression of SYP and PSD 95, although increased expression of PSD95 failed to reach statistical significance (see Additional file 2: Fig. S1a–c). Morphological analysis of the CA1 pyramidal neuron dendrites in the hippocampal slices demonstrated that diabetes reduced spine number, which was reversed by SIRT3 overexpression (Fig. 2b, c). Electron microscopy studies of the hippocampus CA1 region revealed that SIRT3 reversed diabetes-induced synapse loss (Fig. 2d, e). Furthermore, Nissl staining demonstrated that diabetes significantly reduced CA1 hippocampal neurons which was reversed by SIRT3 overexpression (Fig. 2f, g). TUNEL assays indicated that SIRT3 overexpression significantly reduced the proportion of apoptotic cells in the hippocampal CA1 region of diabetic mice (see Additional file 2: Fig. S2a, b). Western blotting analysis showed that SIRT3 overexpression enhanced the expression of anti-apoptotic Bcl2 and reduced expression of proapoptotic Bax and cleaved caspase-3 (see Additional file 2: Fig. S2c, d). Taken together, these data demonstrate that SIRT3 can rescue hippocampal neurons from diabetes-induced injury (Additional file 1).

As synaptic plasticity provides a cellular basis for learning and memory, we next tested whether up-regulation of SIRT3 could improve diabetes-induced cognitive deficit. In the NOR test, SIRT3 overexpression significantly increased the novel object preference of diabetic mice during the test phase, while recognition was comparable among control, SIRT3-overexpressing control, STZ and SIRT3-overexpressing STZ groups during the trial phase (Fig. 2h, i). In the MWM test, SIRT3 overexpression improved diabetes-associated spatial learning deficits as evidenced by reduced latency in finding the submerged escape platform during the 5 consecutive days of training and spatial memory deficits as measured by more frequent crossings of the former platform location and increased time spent in the former platform quadrant (target quadrant) during the probe test on the 7th day (Fig. 2j–n). Contrastingly, there was no significant difference in the swimming speed among the four groups, excluding a contribution of motor deficits (Fig. 2o). These data demonstrate that up-regulating SIRT3 can attenuate diabetes-induced cognitive deficits in mice. Collectively, our study demonstrates that SIRT3 ameliorates hippocampal injuries and cognitive deficits in diabetic mice.

### SIRT3 mitigates mitochondria Ca^2+^ overload-induced mitochondrial dysfunction and apoptosis under HG conditions

**Fig. 3:**
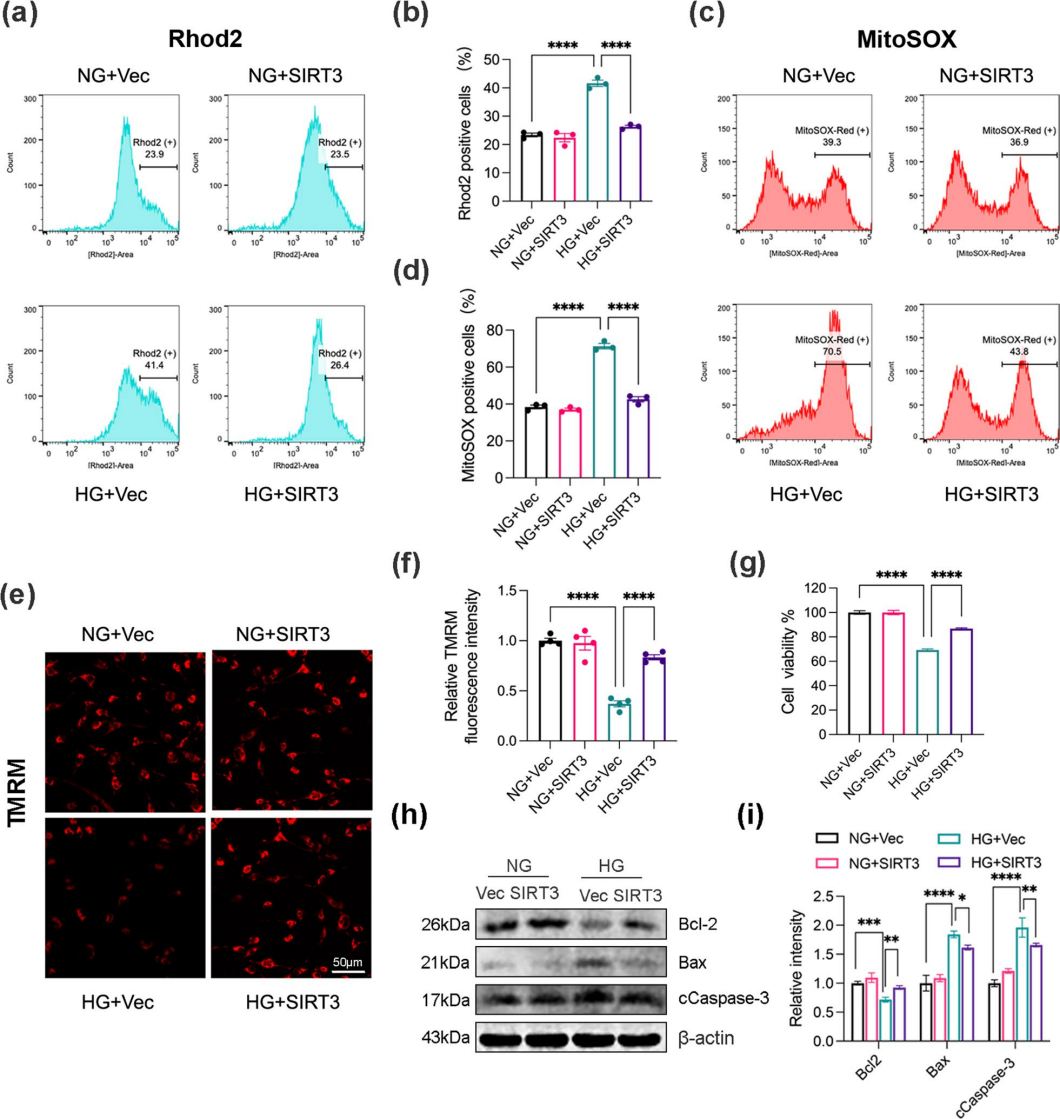
SIRT3 rescues mitochondria Ca^2+^-induced mitochondrial dysfunction and apoptosis under HG condition. **a**, **b** Representative flow cytometry histograms and quantitative analysis of Rhod2-positive SH-SY5Y cells. Rhod-2 AM staining was performed to measure mitochondrial Ca^2+^ levels. N = 3. **c**, **d** Representative flow cytometry histograms and quantitative analysis of MitoSOX-positive cells in SH-SY5Y cells. Rhod-2 AM staining was used to detect mitochondrial ROS (mtROS). N = 3. **e**, **f** Representative confocal imaging of mitochondrial membrane potential (MMP) using TMRM staining. Analysis of MMP by measuring TMRM fluorescence signal. N = 4. **g** The viability of cells was detected by CCK8 assay. N = 3. **h**, **i** Western blot analysis of pro- and anti-apoptotic proteins. cCaspase-3, Cleaved Caspase-3. β-actin was used as the loading control. N = 3. Data were expressed as mean ± SD, *p < 0.05, **p < 0.01, ***p < 0.001, ****p < 0.0001

Abnormal mitochondrial calcium homeostasis induces mitochondrial dysfunction and apoptosis, leading to cognitive disorders. Rhod-2 AM staining and flow cytometry indicated that HG induced mitochondrial calcium overload, which was reversed by SIRT3 overexpression (Fig. 3a, b). Next, we monitored mtROS production was monitored using MitoSOX Red staining. Representative histograms and flow cytometry analysis for mtROS levels suggested that SIRT3 suppressed HG-induced mtROS accumulation (Fig. 3c, d) and restored MMP in HG-treated cells, as illustrated by increased TMRM fluorescence signals (Fig. 3e, f). Furthermore, CCK-8 assay illustrated that SIRT3 reversed the inhibitory effect of HG on cell proliferation (Fig. 3g). Western blotting analysis of apoptosis-related proteins revealed that SIRT3 up-regulation increased the levels of Bcl2 but decreased the levels of Bax and cleaved caspase 3 levels, indicating its anti-apoptotic role in an HG environment (Fig. 3h, i). Overall, these findings demonstrated that SIRT3 favours cell survival by attenuating mitochondrial calcium overload induced mitochondrial dysfunction and apoptosis under HG conditions.

### SIRT3 mitigates excessive MAM formation under HG condition

**Fig. 4:**
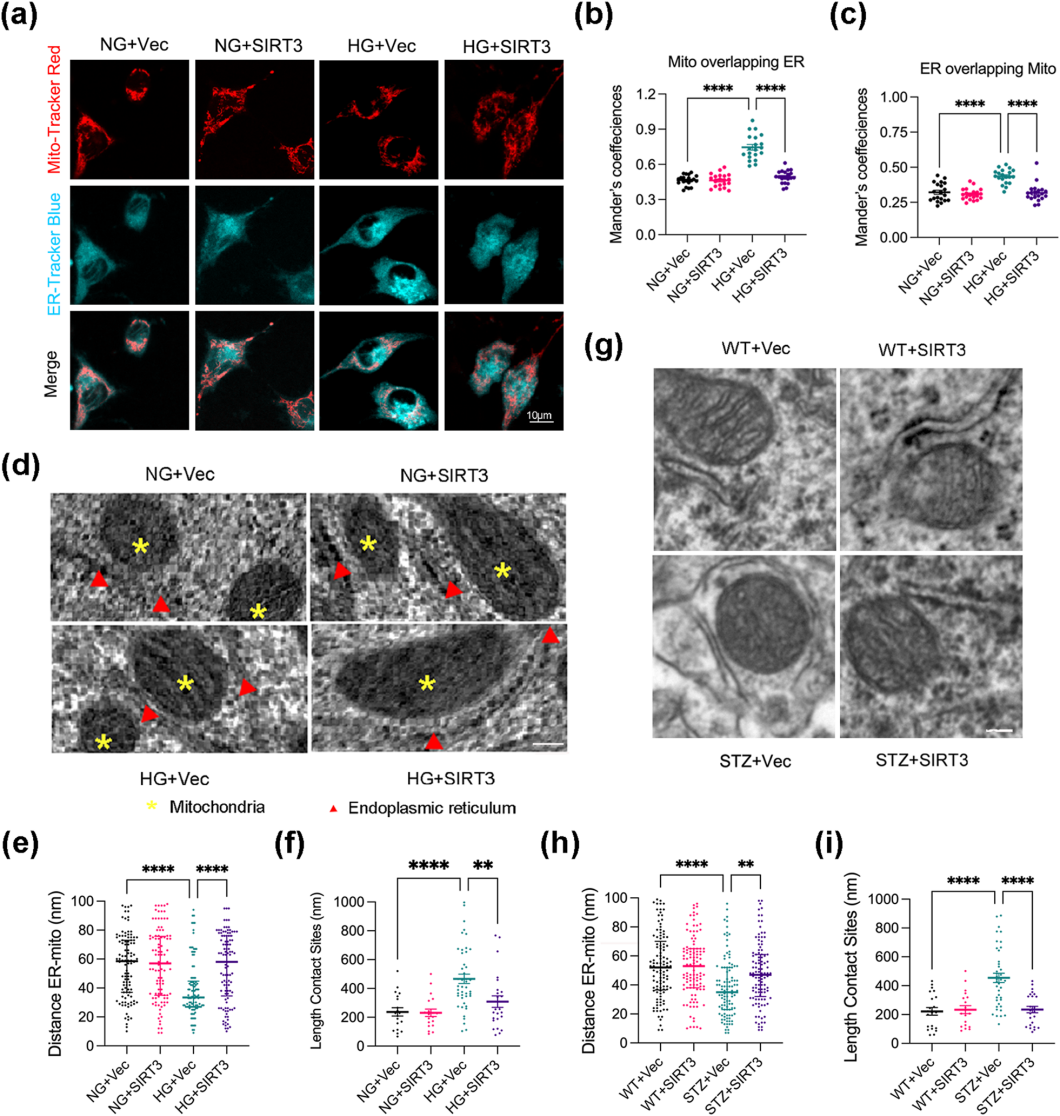
SIRT3 prevents HG-induced MAM formations. **a** Representative confocal images showing the co-localization of ER (ER-Tracker Blue-White DPX staining) and mitochondria (Mito-Tracker Red CMXRos staining). **b**, **c** Mander’s overlap coefficients based on confocal imaging. N = 20 images for each group. **d** Representative electron micrograph images showing the interactions between ER and mitochondria, scale bar = 100 nm. **e**, **f** Quantitative analysis of the distance between the mitochondria and ER (**e**) and the contact length (**f**), N ≥ 100 contacts from 25 images for each group. **g** Representative electron micrographs of ER-mitochondria contacts in the hippocampus, scale bar = 100 nm. **h**, **i** Quantitative analysis of the distance between the mitochondria and ER (**h**) and the length of contacts (**i**). N ≥ 100 contacts from 4 mice per group. The distance between the mitochondria and ER are presented as the median and range (25th–75th percentile), the contact length are presented as mean ± SEM and other data are presented as the mean ± SD, **p < 0.01, ****p < 0.0001

MAMs are considered crucial mediators of Ca^2+^ transportation [30]. As imbalances in Ca^2+^ homeostasis occurs under HG conditions, we next explored whether SIRT3 is involved in regulating MAM coupling. First, we infected the SH-SY5Y cells with SIRT3 lentivirus. The overexpression efficiency of SIRT3 was confirmed via immunobotting and fluorescence images (see Additional file 2: Fig. S3a–c). To investigate the effect of hyperglycemia on MAMs, we assessed the mitochondria–ER contacts after HG exposure using confocal microscopy to visualise mitochondria and ER labelled with Mito-Tracker Red CMXRos and ER-Tracker Blue-White DPX, respectively (Fig. 4a–c). Quantitative analysis indicated higher co-localisation of mitochondria with ER was greater upon HG stimulation than that observed in the control group, evidenced by increased Manders overlap coefficients. However, SIRT3 overexpression reversed the close contact between the ER and mitochondria induced by HG.

To further verify these results, we studied MAM coupling at the ultrastructural level via ΤΕΜ (Fig. 4d–f). Quantitative analysis revealed that the distance between mitochondria and ER was significantly decreased, while the length of MAMs was elongated in the HG group. SIRT3 overexpression significantly increased the distance between mitochondria and ER and decreased contact length, reducing the association of the ER with mitochondria in the HG group. Consistently, analysis of TEM images also demonstrated that diabetes induction dramatically increased MAM formation in the hippocampus, and that this effect was reversed by SIRT3 overexpression (Fig. 4g–i). Together, these data indicate that SIRT3 overexpression blocks the excessive ER–mitochondrial association in vitro and in vivo.

### SIRT3 suppresses the interaction between VDAC1, GRP75 and IP3R

**Fig. 5:**
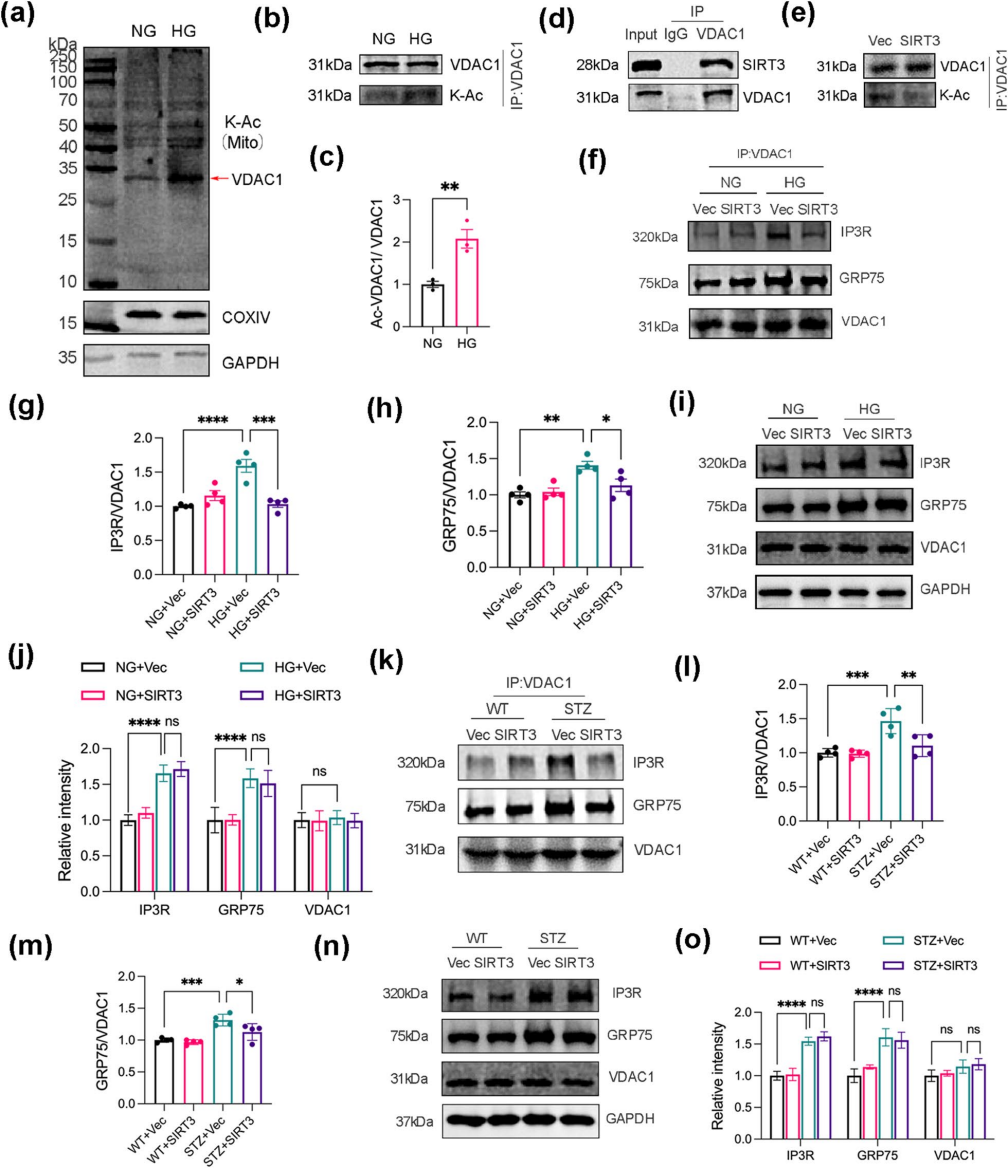
SIRT3 diminishes the interaction between VDAC1, GRP75 and IP3R. **a** Lysine acetylation detected in a mitochondrial extract from SH-SY5Y cells by western blot using an anti-pan acetyl-lysine antibody. K-Ac, acetyl-lysine. COXIV and GAPDH were used as markers for mitochondria and cytoplasm, respectively. **b**, **c** VDAC1 acetylation status examined by immunoprecipitation with an anti-VDAC1 antibody followed by western blot analysis using an anti-acetylated-lysine antibody. The ratios of acetylated VDAC1/total VDAC1 were shown in **c**. K-Ac, acetyl-lysine. N = 3. **d** Co-immunoprecipitation (Co-IP) assay showing the interaction between SIRT3 and VDAC1. **e** Western blots of acetylated VDAC1 by immunoprecipitation in SH-SY5Y cells. K-Ac, acetyl-lysine. **f**–**h** Western blots of IP3R, GRP75, and VDAC1 in VDAC1 immunoprecipitation in SH-SY5Y cells (**f**); Quantification for the expression of IP3R and GRP75 normalised to VDAC1 (**g**, **h**); N = 4. **i**, **j** Western blots (**i**) and quantification analysis (**j**) of IP3R, GRP75, and VDAC1. GAPDH was used as the loading control, N = 4. **k**, **m** Western blot analysis of IP3R, GRP75, and VDAC1 expression in VDAC1 immunoprecipitate from hippocampal tissue. N = 4 mice per group. **n**, **o** Western blot analysis of IP3R, GRP75, and VDAC1 expression in the hippocampus. GAPDH was used as the loading control. Data were expressed as mean ± SD, N = 4.*p < 0.05, **p < 0.01, ***p < 0.001, ****p < 0.0001

To elucidate the mechanism of SIRT3 in regulating MAM coupling, we detected mitochondrial acetylation profiles via an immunoblotting assay using polyclonal antibodies against acetyl-lysine (K-Ac). We found that the protein acetylation profile was globally elevated in the mitochondrial fraction (Fig. 5a). Intriguingly, we observed significantly up-regulated acetylated protein bands near 35 kDa. Based on the existing literature, we identified this band as VDAC1, a major component of the outer mitochondrial membrane [13, 31]. An immunoprecipitation experiment using an anti-VDAC1 antibody was performed to verify this band (Fig. 5b, c). Although the acetylation level of VDAC1 was significantly increased, we observed no changes in VDAC1 protein level (see Additional file 2: Fig. S4a, b). Co-immunoprecipitation revealed that SIRT3 might physically interact with VDAC1 and potentially act as a deacetylase for VDAC1 (Fig. 5d). Given the fact that VDAC1 is expressed in the outer mitochondria membrane, the cytosolic SIRT3 fraction might deacetylate VDAC1 instead of mitochondrial SIRT3 [32]. Consistently, SIRT3 overexpression was found to promote VDAC1 deacetylation in vivo and vitro (Fig. 5e, Additional file 2: Fig. S5a).

As acetylation modification may change the biological function of the protein, we proposed that SIRT3-mediated VDAC1 deacetylation may affect its interaction with GRP75 and IP3R and prevent excessive MAM formations. Our results confirmed this hypothesis. The co-immunoprecipitation data showed that SIRT3 abrogated the enhanced interactions among IP3R, GRP75 and VDAC1 in HG-treated SH-SY5Y cells (Fig. 5f–h). Whereas HG stimulated the expression of GRP75 and IP3R but did not affect the expression of VDAC1 (Fig. 5i, j). This excludes the possibility that SIRT3 altered the assembly of IP3R–GRP75–VDAC1 complexes by directly affecting the expression of these proteins. Consistently, SIRT3 suppressed IP3R–GRP75–VDAC1 complex formation in the hippocampi of diabetic mice (Fig. 5k–m) without altering total protein expression (Fig. 5n, o). Taken together, these results suggest that SIRT3 suppressed HG-induced increase in the interaction among IP3R, GRP75 and VDAC1, which might functions via VDAC1 deacetylation.

### SIRT3 activation by honokiol alleviates MAM formation and exerts a neuroprotective effect in diabetic mice

**Fig. 6:**
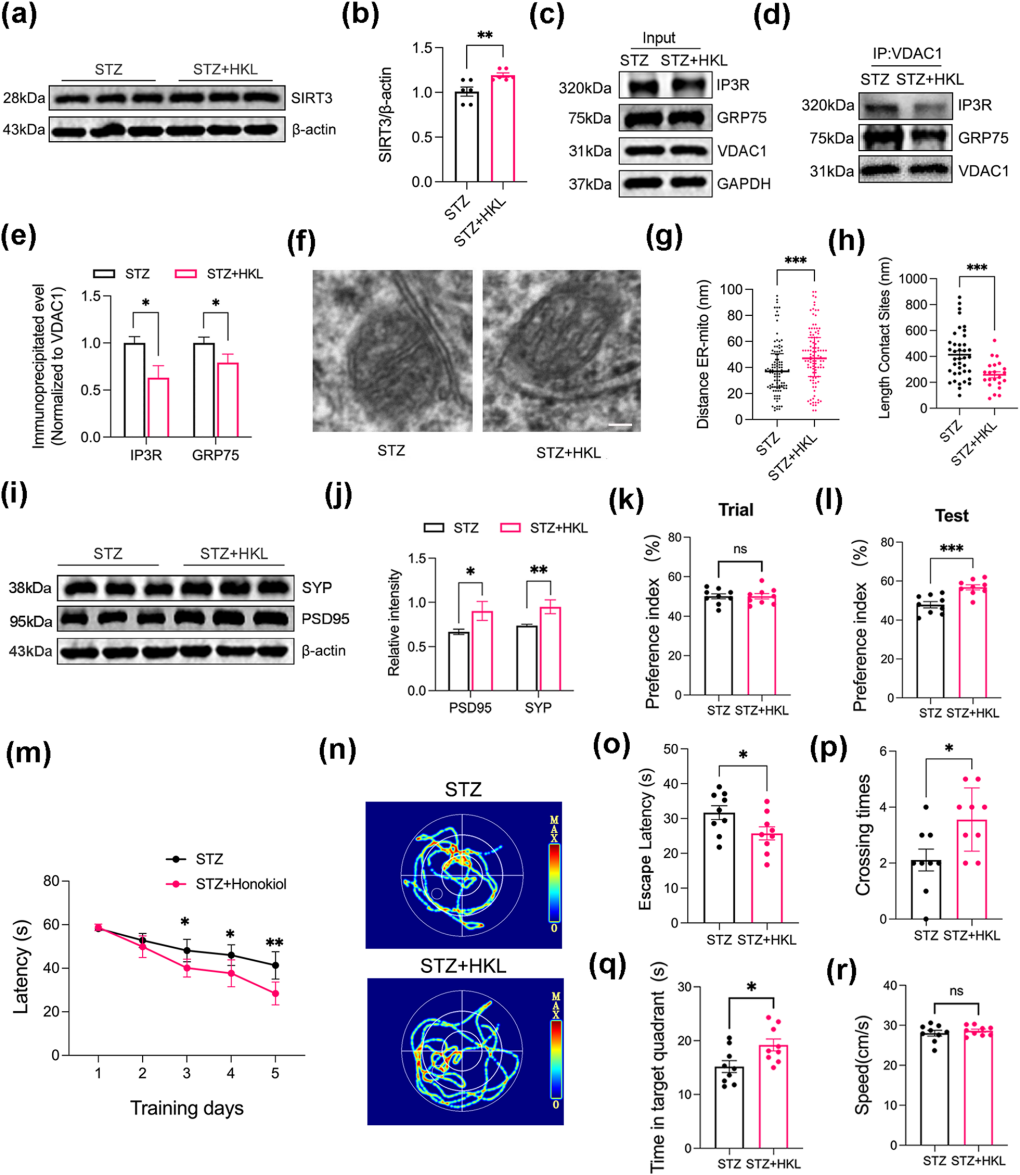
Activation of SIRT3 by honokiol prevents MAM formation and exerts neuroprotective effect in diabetic mice. **a**,** b** Western blot analysis of SIRT3 expression in the hippocampus from diabetic mice with or without honokiol (HKL) treatment. Diabetic mice received intraperitoneal injections of HKL (40 mg/kg/day) for 8 weeks. β-actin served as the loading control. N = 6 mice/group. (c-e) Western blot analysis of IP3R, GRP75, and VDAC1 expression in VDAC1 immunoprecipitate from hippocampal tissue. N = 3 mice/group. **f** Representative electron micrograph images of ER-mitochondria contacts in the hippocampus of diabetic mice with and without honokiol (HKL). Scale bar = 100 nm. **g**, **h** Quantitative analysis of the distance between the mitochondria and ER (**g**) and the length of contacts (**h**). N ≥ 100 contacts from 4 mice per group. **i**, **j** Western blot analysis of Synaptophysin (SYP) and postsynaptic density protein 95 (PSD95) expression in the hippocampus. β-actin was used as the loading control. N = 3 mice/group. (**k**, **l**) Preference index during training and testing phase in novel object recognition (NOR) test. N = 9 mice/group. **m**-**r** Spatial learning and memory as measured by the Morris water maze (MWM) test. Latency to find the hidden platform during 5 consecutive training days (**m**). Representative swimming paths of each group during the test phase (**n**). Escape latency to reach the absent platform (**o**), platform crossing times (**p**), duration in the target quadrant (**q**), and average swimming speed (**r**) during the probe test on the 7th day. N = 9 mice/group. Data were expressed as mean ± SD for (**a**–**e**, **i**, **j**), mean ± SEM for (**h**, **k**–**r**), and median and range (25th–75th percentile) for (**f**, **g**), *p < 0.05, **p < 0.01, ***p < 0.001

We showed that SIRT3 alleviated MAM formations under HG conditions. Furthermore, we examined if the small molecule SIRT3 agonist honokiol (HKL) could replicate the effects of SIRT3 overexpression in diabetic mice. Indeed, co-immunoprecipitation assay results showed that enhancement of SIRT3 expression by HKL (Fig. 6a, b) decreased the interaction between IP3R, GRP75 and VDAC1 in diabetes model mice (Fig. 6c–e). Also, TEM analysis revealed fewer ER–mitochondria contacts in diabetic mice after HKL treatment (Fig. 6f–h). These results demonstrate that pharmacological activation of SIRT3 by HKL treatment can reduce excessive MAM formation and protect hippocampal neurons from diabetes-induced injury in hippocampus.

Futhermore, we investigated if HKL treatment could also restore synaptic function and cognition in diabetic mice. Indeed, HKL significantly increased hippocampal expression of the presynaptic marker protein SYP and the postsynaptic marker protein PSD95 (Fig. 6i, j). Treatment with HKL also restored NOR memory (Fig. 6k, l) as well as spatial learning and memory (Fig. 6m–r) in diabetic mice.

## Discussion

**Fig. 7:**
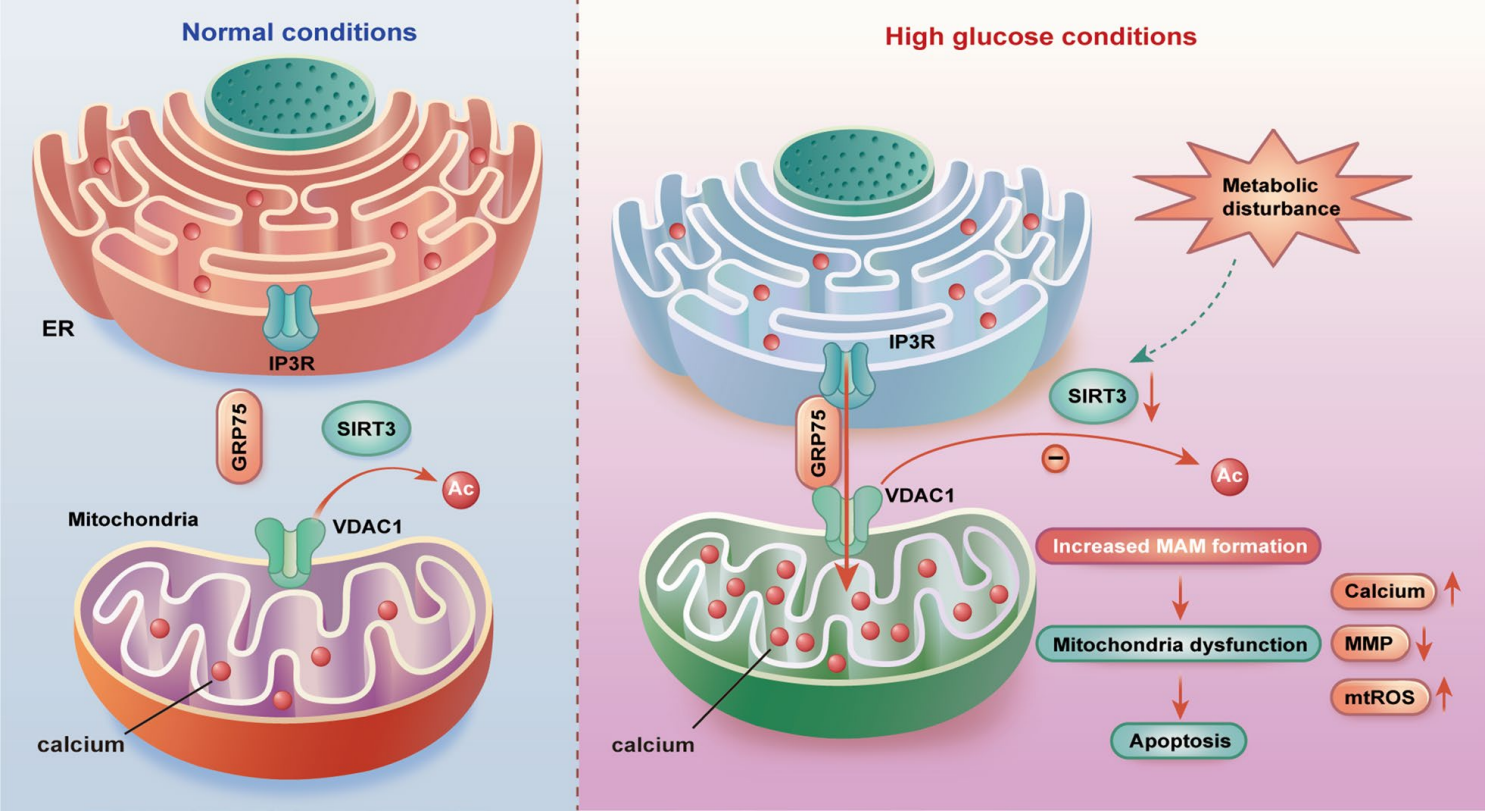
Schematic illustration of the underlying mechanism of SIRT3 (Sirtuin 3) in regulating MAM coupling. Under normal conditions, SIRT3 is highly expressed and promotes VDAC1 deacetylation. Hypoacetylated VDAC1 binds to cytoplasmic chaperone GRP75 and tethers ER-resident inositol IP3R with low affinity. During hyperglycemia, SIRT3 expression is inhibited, leaving VDAC1 in a hyperacetylated state. Hyperacetylated VDAC1 displays significantly stronger interaction with GRP75 and IP3R, resulting in increased MAM formation, mitochondrial dysfunction and apoptosis

In this study, we observed a dramatic reduction in SIRT3 expression in the hippocampal tissues of diabetic mice and HG-exposed SH-SY5Y cells. SIRT3 overexpression rescued hippocampus injuries and cognitive impairment in diabetic mice by alleviating mitochondrial dysfunction and apoptosis. Mechanistically, we revealed that HG stimulated abbarent MAM formations and genetic up-regulation of SIRT3 could redefine MAM formation via decreasing IP3R–GRP75–VDAC1 interactions. Lastly, administration of the pharmacological SIRT3 activator honokiol exerted therapeutic effects comparable to our genetic intervention in diabetic mice. Hence, our findings provide a novel pathologic and therapeutic understanding of SIRT3 in diabetes-associated cognitive impairment (Fig. 7).

SIRT3, a key deacetylase for mitochondrial proteins, has been well-estabilshed as an important regulator of mitochondrial functions, including energy metabolism, apoptosis and calcium exchange [33–35]. Recent evidence has indicated that SIRT3 deficiency or functional abnormality is closely associated with the initiation and progression of various disorders, such as diabetes, cardiovascular diseases, neurodegenerative diseases and cancer [22, 36, 37]. Zheng et al. reported that in hyperglycemic intracerebral haemorrhage rats, SIRT3 ameliorates mitochondrial dysfunction by improving ATP generation and mitochondrial biogenesis [19]. Yin et al. also reported that SIRT3 overexpression increased deacetylation activity, and rescued mitochondrial function including the enhanced ATP production in an Alzheimer's disease model [38]. In the present study, we identified a dramatic reduction of SIRT3 expression in high glucose-treated SH-SY5Y cells and hippocampal tissues from diabetic mice. Furthermore, SIRT3 overexpression protected against hippocampal injuries and cognitive impairment in diabetic mice.

MAM is a site of physiological communication between the mitochondria and ER, promoting interaction between these two organelles and regulating physiological activities in cells [39, 40]. However, aberrant MAM formation might cause mitochondrial dysfunction via mitochondria calcium overload, leading to cell apoptosis [41]. Notably, several recent studies demonstrated that disrupting aberrant MAM formation can reverse these pathological processes. For instance, Lee et al. demonstrated that inhibiting ER-mitochondria contacts by urolithin A ablated mitochondrial calcium overload and mtROS accumulation under HG exposure by suppressing the expression of transglutaminase 2, a regulatory element of the IP3R–VDAC1 bridge [13]. Wu et al. also reported that ablation of FUN14 domain containing 1 (Fundc1) in cardiomyocytes prevented aberrant MAM formation and increased mitochondrial Ca^2+^ [42]. Here, we found that the contact sites between the ER and mitochondria were enhanced under hyperglycemic conditions. Furthermore, we demonstrated SIRT3 overexpression ameliorated aberrant MAM formation and suppressed HG-induced mitochondrial calcium overload, mtROS accumulation and ensuing cytotoxicity.

MAM formation depends on proteins located on membranes of ER and mitochondria, which interact directly or indirectly by forming multiprotein-tethering complexes [43]. IP3R–GRP75–VDAC1 tripartite complexes, known as critical Ca^2+^ channel complexes at the MAMs interface, are responsible for the transfer of excessive calcium from ER to mitochondria [44, 45]. In this study, we found that SIRT3 overexpression suppressed the  IP3R–GRP75–VDAC1 interactions. A growing number of studies have shown that protein levels or post-translational modifications affect the biological functions of MAMs. Birgit Honrath revealed that overexpressing GRP75 enhanced IP3R1–VDAC1 interactions [46]. Charles Betz reported that Akt phosphorylates the IP3R and regulates MAM integrity [47]. Post-translational modifications of VDAC1, a multi-functional protein anchored in the mitochondrial outer membrane, are essential for modulating its biochemistry and interactions [48–51]. In this study, We found that SIRT3 physically interacts with VDAC1 and promotes its deacetylation. Thus, we proposed that SIRT3 might mediate VDAC1 deacetylation and resultantly suppress the formation of IP3R–GRP75–VDAC1 complexes. However, the effect of VDAC1 deacetylation and its possible deacetylated sites need to be further explored.

Overall, these findings support SIRT3 as a valid target for the preventing excessive MAM formation and ensuing diabetic dementia. To test the clinical translation of this idea, we evaluated the therapeutic efficacy of honokiol, a SIRT3 activator, in STZ-induced diabetic model mice. Consistent with our genetic intervention, activation of SIRT3 by honokiol treatment exhibited a good therapeutic effect, as evidenced by reduced synaptic loss and significant improvements in NOR and MWM tests. Honokiol is a natural phenolic compound extracted from Magnolia grandiflora with reported anti-inflammatory, anti-oxidant, and anti-depressive effects and a good biosafety profile [52]. More importantly, honokiol can penetrate the blood–brain barrier [53]. Previous studies have shown that honokiol protected hippocampal neurons and improved learning and memory in several animal models of AD [54–56]. Our findings reveal a possible mechanism underlying this therapeutic effect and provide additional evidence for SIRT3 activators as potential therapeutic agents for diabetic dementia patients.

This study has several strengths, making significant in this field. Notably, we conducted both in vivo and in vitro experiments. We performed multiple assays, such as co-immunoprecipitation, TEM analysis and ER and mitochondrial co-localisation analysis, to explore the role of SIRT3 in regulating MAMs coupling. Βased on the genetic manipulation, we further tested a pharmacological strategy. However, our study still has some limitations that warrants further investigation. First, this study might have a potential bias due to the limited sample size and unblinded analysis of some experiments. Second, the effect of SIRT3-mediated VDAC1 deacetylation on the interactions with other proteins aside from GRP75 and IP3R remains unclear. Finally, we cannot exclude the contributions of other mitochondria sirtuins, such as SIRT4 and SIRT5, which might affect MAM coupling.

## Conclusion

In summary, our study demonstrates a previously unknown relationship between SIRT3 and MAM coupling in diabetes-induced cognitive dysfunction. In addition, we provide additional evidence that SIRT3 agonists like honokiol may be useful for preventing and treating diabetic dementia. However, future therapeutic perspectives should consider the potential limitations and implications of targeting SIRT3 and MAMs, such as potential side effects and long-term efficacy. Besides, considering that MAM coupling is implicated as an earlier event in the process of Alzheimer’s disease-type pathologies, further investigation is required to understand the mechanisms underlying aberrant MAM formation and the potential role of SIRT3 in other types of cognitive impairment.

## Supplementary Information

**Additional file 1:** Key resources table.

**Additional file 2:** **Fig. S1.** SIRT3 promotes the expression of Synaptic Proteins in the hippocampus of diabetic mice. **a**-**c** Western blot analysis of Synaptophysin (SYP) and postsynaptic density protein 95 (PSD95) expression in the hippocampus. **Fig. S2.** SIRT3 reduces neuronal apoptosis in the hippocampus of diabetic mice. **Fig. S3.** SIRT3 overexpression in SH-SY5Y cells. **Fig. S4.** HG does not alter VDAC1 protein level in SH-SY5Y cells. **Fig. S5.** SIRT3 promotes VDAC1 deactylation in hippocampus from diabetes-induced mice.

## Data Availability

The datasets supporting the conclusions of this article are included within the article and its additional file. For any further data requests, please contact the corresponding authors.

## Abbreviations

HKL: Honokiol
GRP75: Glucose-regulated protein 75
ER: Endoplasmic reticulum
IP3R: Tripartite 1,4,5-trisphosphate receptor
MAMs: Mitochondria-associated membranes
MMP: Mitochondrial membrane potential
mtROS: Mitochondrial reactive oxygen species
PSD95: Postsynaptic density protein 95
SIRT3: Sirtuin 3
SYP: Synaptophysin
VDAC1: Voltage-dependent anion channel 1
