# Supplementary material for: SIRT3 ameliorates diabetes-associated cognitive dysfunction via regulating mitochondria-associated ER membranes
Source: J Transl Med. 2023 Jul 22;21:494. doi: 10.1186/s12967-023-04246-9 (PMC10362714; doi:10.1186/s12967-023-04246-9)
Supplement: Supplementary file 1 — Additional file 1: Key resources table. [file 12967_2023_4246_MOESM1_ESM.docx]

Key Resources Table

| REAGENT or RESOURCE | SOURCE | IDENTIFIER | | |
| --- | --- | --- | --- | --- |
| Antibodies | | | |  |
| Rabbit monoclonal Anti-SIRT3 | Cell Signaling Technology | 5490S |  |  |
| Rabbit monoclonal Anti-IP3R | Abcam | ab108517 |  |  |
| Rabbit polyclonal Anti-VDAC1 | Proteintech | 55259-1-AP |  |  |
| Rabbit polyclonal Anti-GRP75 | Proteintech | 14887-1-AP |  |  |
| Rabbit polyclonal Anti-Pan Acetyl-Lysine | Abclonal | A2391 |  |  |
| Rabbit polyclonal Anti-COXIV | Proteintech | 11242-1-AP |  |  |
| Mouse monoclonal Anti-Bcl2 | Cell Signaling Technology | 15071 |  |  |
| Rabbit polyclonal Anti-Bax | Proteintech | 50599-2-Ig |  |  |
| Rabbit monoclonal Anti-cCaspase-3 | Cell Signaling Technology | 9664 |  |  |
| Rabbit polyclonal Anti-Synaptophysin | Proteintech | 17785-1-AP |  |  |
| Rabbit polyclonal Anti-PSD95 | Proteintech | 20665-1-AP |  |  |
| Mouse monoclonal Anti-GAPDH | Proteintech | 60004-1-Ig |  |  |
| Mouse monoclonal Anti-Beta Actin | Proteintech | 66009-1-Ig |  |  |
| Anti -EGFP Mouse mAb | Servicebio | GB12602 |  |  |
| Goat anti-Mouse HRP | Proteintech | SA00001-1 |  |  |
| Goat anti- Rabbit HRP | Proteintech | SA00001-2 |  |  |
| Alexa Fluor 488-conjugated goat anti-rabbit IgG | Servicebio | GB25303 |  |  |
| Bacterial and virus strains | | | |  |
| AAV 2/9 | OBiO | N/A |  |  |
| Lentivirus | OBiO | N/A |  |  |
| Reagent or Resouce |  |  |  |  |
| DMEM, High glucose | GIBCO | 11965092 |  |  |
| Fetal Bovine Serum | Every Green | 11011-8611 |  |  |
| BCA protein assay kit | Beyotime | P0012S |  |  |
| Protein A+G Agarose | Beyotime | P2019 |  |  |
| Mito-Tracker Red CMXRos | Beyotime | C1049B |  |  |
| ER-Tracker Blue-White DPX | Yeason | 40761ES50 |  |  |
| Rhod-2 AM | Yeason | 40776ES72 |  |  |
| Tetramethylrhodamine, ethyl ester (TMRM) | Beyotime | C2001S |  |  |
| MitoSOX^TM^ Red | Thermo Fisher Scientific | M36008 |  |  |
| Cell Counting Kit-8 | Servicebio | G4103-1ML |  |  |
| Mitochondria Isolation and Protein Extraction Kit | Proteintech | PK10016 |  |  |
| Chemiluminescence detection kit | Biosharp | BL520A |  |  |
| Streptozocin | Solarbio | S8050 |  |  |
| Honokiol | MedChemExpress | HY-N0003 |  |  |
| FD Rapid GolgiStain^TM^ Kit | FD neurotechnology | PK401 |  |  |
| Nissl staining solution | Servicebio | G1036 |  |  |
| One-step TUNEL In Situ Apoptosis Kit | Elabscience | E-CK-A320 |  |  |
