# Supplementary material for: SIRT3 ameliorates diabetes-associated cognitive dysfunction via regulating mitochondria-associated ER membranes
Source: J Transl Med. 2023 Jul 22;21:494. doi: 10.1186/s12967-023-04246-9 (PMC10362714; doi:10.1186/s12967-023-04246-9)
Supplement: Supplementary file 2 — Additional file 2: Fig. S1. SIRT3 promotes the expression of Synaptic Proteins in the hippocampus of diabetic mice. a-c Western blot analysis of Synaptophysin (SYP) and postsynaptic density protein 95 (PSD95) expression in the hippocampus. Fig. S2. SIRT3 reduces neuronal apoptosis in the hippocampus of diabetic mice. Fig. S3. SIRT3 overexpression in SH-SY5Y cells. Fig. S4. HG does not alter VDAC1 protein level in SH-SY5Y cells. Fig. S5. SIRT3 promotes VDAC1 deactylation in hippocampus from diabetes-induced mice. [file 12967_2023_4246_MOESM2_ESM.docx]

**
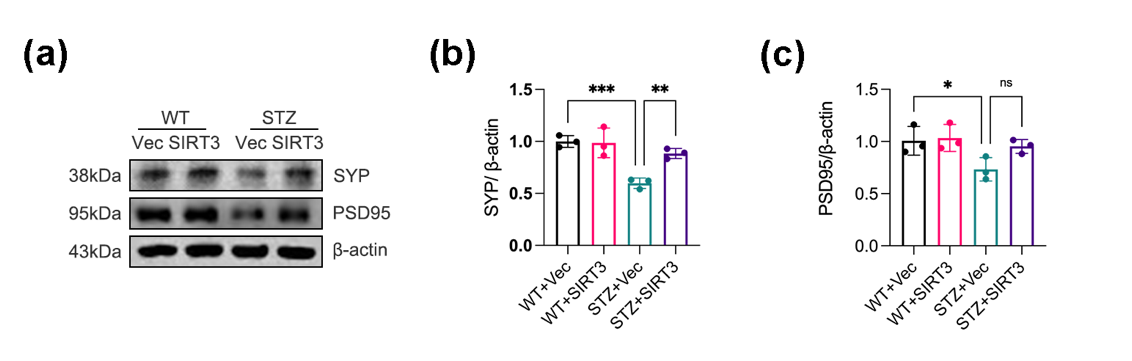
**

**Supplementary Fig. 1 SIRT3 promotes the expression of Synaptic Proteins in the hippocampus of diabetic mice.**

(a-c) Western blot analysis of Synaptophysin (SYP) and postsynaptic density protein 95 (PSD95) expression in the hippocampus. β-actin was used as the loading control. N = 3 mice /group. Data were expressed as mean ± SD, *p < 0.05，**p < 0.01, ***p < 0.001


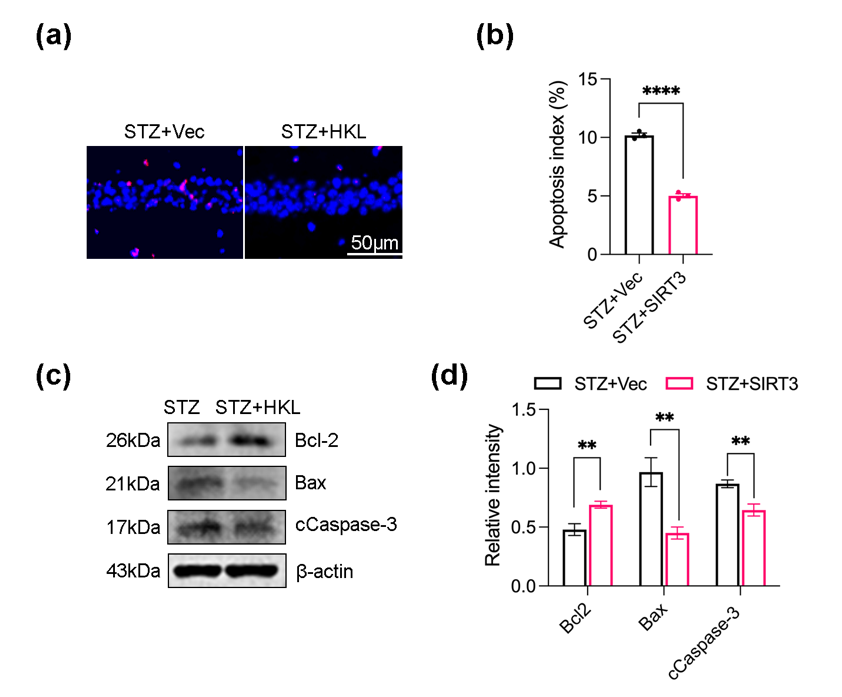


**Supplementary Fig. 2 SIRT3 reduces neuronal apoptosis in the hippocampus of diabetic mice.**

(a) Representative TUNEL staining (red) of the hippocampal CA1 region. Nuclei were counterstained with DAPI (blue).(b)Proportion of TUNEL-positive cells among DAPI-positive cells. N=3 mice/ group. (c-d) Western blot analysis of apoptosis-related proteins in the hippocampus. cCaspase-3, cleaved Caspase-3. N = 3 mice /group. Data were expressed as mean ± SD , **p < 0.01, ****p < 0.0001

**
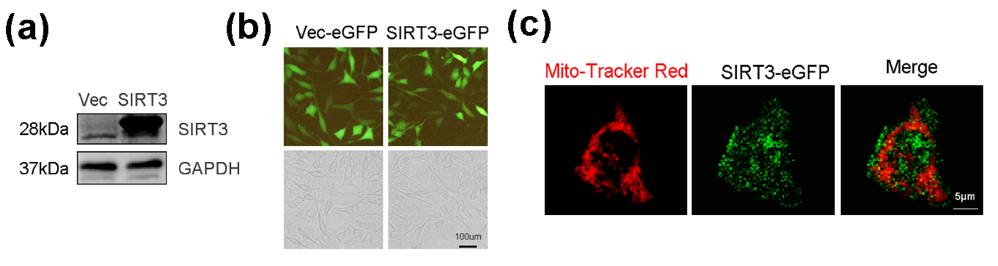
**

**Supplementary Fig. 3 SIRT3 overexpression in SH-SY5Y cells**

(a)Western blotting showing SIRT3 protein level in SH-SY5Y cells infected with Lenti-CMV-eGFP. (b) Representative fluorescence images confirming the efficiency of lentivirus infection. (c) Representative confocal images of SH-SY5Y cell expressing Lenti-CMV-SIRT3-eGFP. Mitochondrial content was visualized using Mito-Tracker Red staining.

**
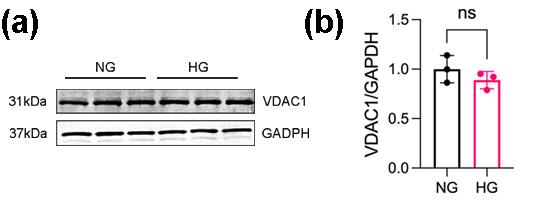
**

**Supplementary Fig. 4 HG does not alter VDAC1 protein level in SH-SY5Y cells.**

(a-b) Western blot analysis of VDAC1 protein expression levels in SH-SY5Y cells. N=3. Data were expressed as mean ± SD.


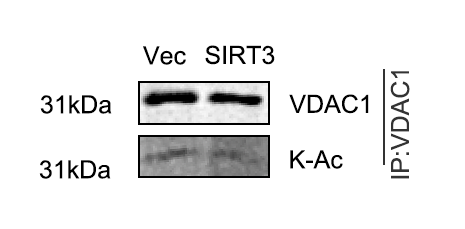


**Supplementary Fig. 5 SIRT3 promotes VDAC1 deactylation in hippocampus from diabetes-induced mice.**

a.Western blots of acetylated VDAC1 by immunoprecipitation in hippocampus. K-Ac, acetyl-lysine.
